# Return to Work of Healthcare Workers after SARS-CoV-2 Infection: Determinants of Physical and Mental Health

**DOI:** 10.3390/ijerph19116811

**Published:** 2022-06-02

**Authors:** Maddalena Grazzini, Lucrezia Ginevra Lulli, Nicola Mucci, Diana Paolini, Antonio Baldassarre, Veronica Gallinoro, Annarita Chiarelli, Fabrizio Niccolini, Giulio Arcangeli

**Affiliations:** 1 Health Direction, Careggi University Hospital, 50134 Florence, Italy; grazzinim@aou-careggi.toscana.it (M.G.); paolinid@aou-careggi.toscana.it (D.P.); niccolinif@aou-careggi.toscana.it (F.N.); 2 Department of Experimental and Clinical Medicine, University of Florence, 50134 Florence, Italy; nicola.mucci@unifi.it (N.M.); antonio.baldassarre@unifi.it (A.B.); giulio.arcangeli@unifi.it (G.A.); 3 Occupational Medicine School, University of Florence, 50134 Florence, Italy; 4 Medical Specialization School of Hygiene and Preventive Medicine, University of Florence, 50134 Florence, Italy; veronica.gallinoro@unifi.it; 5 Occupational Medicine Unit, Careggi University Hospital, 50134 Florence, Italy; chiarellian@aou-careggi.toscana.it

**Keywords:** SARS-CoV-2 infection, healthcare workers, return to work, aging workforce, gender, sleep alterations, health perception, occupational health, occupational wellbeing, resilience

## Abstract

Introduction. The SARS-CoV-2 pandemic has involved healthcare workers (HCWs) both as caregivers and as patients. This study is a retrospective cross-sectional analysis of the HCWs working in a third-level hospital in Central Italy who were infected with COVID-19 from March 2020 to April 2021. This research aims at identifying the physical and mental health outcomes of HCWs infected with COVID-19 who returned to work after the infection, the determinants of those outcomes, such as age and sex, and the identification of possible vulnerable professional groups. Methods. A questionnaire about the acute illness, the experience of returning to work, and health perceptions after the disease was administered to 427 healthcare workers 3 months after recovering from the SARS-CoV-2 infection. Results. The majority interviewed (84.5%) reported symptoms at the time of the positive test, with no significant differences regarding age or sex, while a significant difference in the mean age was found regarding hospitalization (*p* < 0.001). At 3 months after the infection, females (*p* = 0.001), older workers (*p* < 0.001), and healthcare assistants (*p* < 0.001) were more likely to report persistent symptoms. Sex (*p* = 0.02) and age (*p* = 0.006) influenced the quality of sleep after the infection. At work, the nurses group reported increase in workload (*p* = 0.03) and worse relationships (*p* = 0.028). At 3 months after the infection, female workers perceived worse physical (*p* = 0.002) and mental (*p* < 0.001) health status according to the SF-12. A negative correlation was found between age and PCS score (*p* < 0.001) but not MCS score (*p* = 0.86). A significant difference in PCS score was found between nurses and physicians (*p* = 0.04) and between residents and all other groups (*p* < 0.001). Finally, the group of workers reporting sleep alterations showed lower PCS and MCS scores (*p* < 0.001) and working relationships had an impact on MCS scores (*p* < 0.001). Conclusions. Age, sex, and type of job had an impact on physical and mental outcomes. Organizing specific interventions, also tailored to professional sub-groups, should be a target for healthcare systems to protect and boost the physical and mental health of their workers.

## 1. Introduction

Two years after its beginning, the SARS-CoV-2 pandemic continues to spread all over the world, which is also due to the emergence of new variants of interest and concern [1]. Healthcare systems have rearranged their services to deal with the new medical needs of the population. Healthcare workers (HCWs), at the very heart of the pandemic, faced the dual nature of the disease, both as caregivers and patients, being exposed in both their living and working environments [2]. The healthcare working environment can be considered a complex and multifaceted setting: workers face high physical workloads with long and strenuous shifts and also experience challenging emotional situations [3,4]. The experience of dealing with emergencies can produce physical and mental impairment [5], which could also increase resilience and personal growth [6]. In the first line, HCWs represent a high-risk population, presenting a 24-times higher probability than the general population of contracting the infection [7]. HCWs are a very heterogeneous population, ranging from the very young, with health workers in their 20s, to workers aged 60 and over who may have a fair number of chronic diseases. The usual COVID-19 clinical spectrum in HCWs ranges from asymptomatic to mild forms, as with the general population [8,9], but it can also include severe and fatal infections [10]. The availability of HCWs is crucial, especially during surges, when, due to the high proportion of infected HCWs, hospitals can become understaffed [11], putting more pressure on the remaining personnel and requiring infected workers to return to work as soon as possible. After COVID-19 infection, the return to work and to usual physical capability may not be so easy. In fact, the long COVID phenomenon is emerging, significantly increasing the interval between infection and the return to work [12] due to physical and psychological symptoms caused by the disease [13,14]. As the incidence of COVID-19 among HCWs is high, the impact of persistent disturbances after the acute illness can also have negative effects on the broader healthcare delivery system, resulting in a possible loss of skilled healthcare personnel due to post-COVID-19 disabilities [15]. In fact, the so-called “long COVID” has been recognized as a clinical entity that may cause significant disability, not only in hospitalized patients but also in asymptomatic or mildly symptomatic ones [16]. Moreover, psychological disorders, such as depression, anxiety, and sleep disturbances were frequently reported to be associated with COVID-19 [12,17]. At the moment of returning to work, difficulties can be faced by those who have been affected by COVID-19 and have experienced physical and psychological disturbances as well as the reorganization of work, the loss of productivity, and the reduced ability to work [18]. In this current scenario, it is important to deeply understand the experience of HCWs affected by COVID-19 who return to work, to support their occupational health and well-being [19,20], and also to identify at-risk groups. 

Multiple studies have addressed the issue of HCW workplace stress related to the emergency, but only a few have considered the health outcomes of the HCWs infected by COVID-19. There is a need for shifting the point of view from HCWs as “caregivers” to HCWs as “patients”, from the primary prevention of infection to a focus on the recovery from it. The issue is relevant because of the higher risk of HCWs in contracting COVID-19 in healthcare settings [21]; in Italy, COVID-19 infection has been recognized as an occupational hazard, thus acknowledging the augmented occupational risk. This study describes the mental and physical outcomes of a sample of HCWs infected by SARS-CoV-2 while working in a single, third-level hospital in Italy, both during the acute illness and at the time of their return to work, three months after the infection. Data were collected after the first and second wave of COVID-19, occurring between spring and autumn 2020. In the COVID-19 pandemic, Italy has been one of the most involved countries, especially at the beginning of 2020 [22]. During the time of the research, several measures were taken in Italy to protect public health. In particular, the country passed through long periods of lockdowns (from the first stricter lockdown of spring 2020 to the lighter ones of autumn 2020 and winter 2021) caused by widespread virus circulation, until the vaccination campaign began to provide the expected results. During this period, the pressure on health-care facilities was huge. To provide an example, the intensive care unit (ICU) occupation rate, which was one of the parameters considered by the Italian government in monitoring the impact of COVID-19 on the healthcare system, registered peaks of 30–99% during the period analyzed. Nowadays, in May 2022, after the end of the emergency in Italy, the rate of occupation for ICU beds is less than 10% [23].

With a view to protecting workers’ health in the workplace, identifying the outcomes, in terms of physical and mental health, both during the acute illness and especially at the time when HCWs returned to work, is necessary in order to plan adequate interventions. From a management point of view, preserving and promoting the return to work of a healthy working population is essential to prevent a shortage in personnel with its significant economic and organizational costs. Thus, this research aims to understand what the physical and mental health determinants of a population of HCWs infected by COVID-19 are and to identify additional possibly vulnerable sub-groups. It can be hypothesized that the possible presence of prolonged COVID-19-related disturbances included in the definition of long COVID, along with the psychosocial struggles encountered during the return to work, can affect the wellbeing of HCWs. These health outcomes may be related to biological characteristics, namely age and sex, but also, based on previous research, to the type of job performed and the type of work organization [24].

## 2. Materials and Methods

### 2.1. Data Collection

This study is a single-center, retrospective, cross-sectional analysis of the HCWs affected by COVID-19 working in a third-level hospital with a care capacity of 1200 beds and approximately 6000 employees in Central Italy. Data were collected through active surveillance within the Health Surveillance managed by the Occupational Medicine Unit and carried out by residents in public health and occupational medicine who were part of the hospital team who followed the infected HCWs (i.e., in terms of “active surveillance”). This included an epidemiological investigation to identify close contacts to prevent the spread of the virus in the hospital, the monitoring of symptoms, support in planning swabs and testing, the planning of the return to work, the providing (if requested) of specific sick-leave certifications, and in some cases, the providing of medical examinations before returning to work. Specifically, the data presented were collected through telephone interviews approximately 3 months after the first positive test (average 100.3 days; standard deviation ± 43.1 days). The study population contracted the SARS-CoV-2 infection before being fully vaccinated. The convenience sample was obtained by contacting each HCW who tested positive from March 2020 to April 2021 and including in the survey those who agreed to participate. The data collection was performed within the health surveillance program, according to Italian Legislative Decree D.Lgs. 81/2008, which regulates health and safety in the workplace. All participants agreed to the processing of their personal data and understood that this information was categorized as “sensitive data” and treated in an anonymous and collective way, by scientific methods, and for scientific purposes in accordance with the principles of the Declaration of Helsinki [25]. Having received exhaustive information on the study protocol, all participants gave their informed consent.

### 2.2. Material

Personal data, clinical and epidemiological data related to SARS-CoV-2 infection, and information related to current health status were collected for each HCW interviewed. Regarding the latter, a multipurpose, short-form generic measure of health, composed of 12 items (SF-12; Short Form health survey) [26] and used by ISTAT (Italian National Statistical Institute, Rome, Italy), was administered to examine perception of the individual psychophysical condition. The synthesis of the scores allows for the construction of two indices regarding the state of health: one concerning the physical state (Physical Component Summary—PCS,) and the other the psychological state (Mental Component Summary—MCS). The questionnaire administered to COVID-19-positive HCWs can be found in Supplementary Materials S1. All data were collected in a spreadsheet.

For the research purposes of this study, the professional roles were divided into Nurses (N), for all the healthcare workers in direct contact with patients (e.g., nurses, obstetricians, radiology technicians, neuropathophysiology technicians); Healthcare Assistants (HC.Ass.), including those working in support of nursing activities; Physicians (MD); Resident Physicians (Res.MD); and Other, including those workers not in direct contact with patients (e.g., clerks, drivers, lab personnel).

### 2.3. Statistical Analysis

JASP 0.16 was used for data analysis. Kurtosis and skewness were tested, and the Shapiro–Wilk test revealed nonparametric distribution of the main continuous variables. The sample size was judged adequate considering the overall working population of the hospital. Therefore, the significance level α was set at 0.05. A two-tailed, chi-square test was used to compare the distribution of nominal data. The non-parametric Mann–Whitney U test and Kruskal–Wallis test were used to compare ordinal data between two or more groups; post hoc analysis was performed with Dunn’s test. Kendall’s test was used to correlate continuous variables.

## 3. Results

Data were collected through active surveillance of the infected workers, within the health surveillance managed by the occupational medicine unit. The survey was carried out on the 517 healthcare workers who tested positive for SARS-CoV-2 using RT-PCR on nasopharyngeal swabs from 7 March 2020 to 22 April 2021. A total of 427 (82.6%) participants joined the study, while 90 (17.4%) refused to participate or were lost at follow-up. The survey was administered through a telephone interview by public health and occupational health resident physicians. The presentation of the findings analyzes the sample characteristics, the variables linked to the acute illness phase, and lastly, the characteristics of the HCWs health status 3 months after the infection. Sex, age, and professional sub-groups are the main terms of comparison considered for each variable. 

### 3.1. Sample Characteristics

The characteristics of the final sample and the main variables analyzed are included in Table 1. The final sample was composed of 143 men (33.5%) and 284 women (66.5%), with a mean age of 45 (45.6 for men, 45.2 for women). Of these, 186 were Nurses (N, 43.6%), 104 Healthcare Assistants (HC.Ass. 24.4%), 51 Physicians (MD, 11.9%), 54 Residents physicians (Res. MD, 12.6%), 32 Other (Oth., 7.5%). Between the professional sub-groups there was a significant difference in relation to the mean age (H (4) = 124.05, *p* < 0.001). The professional groups also differed by gender, with a prevalence of female workers in the groups Nurses, Healthcare Assistants and Other, and a prevalence of male workers in the groups Physicians and Resident Physicians, χ^2^ (4, 427) = 45.65, *p* < 0.001.

### 3.2. The Acute Illness

Healthcare workers underwent the SARS-CoV-2 swab for several reasons. Briefly, 160 (37.5%) had contact with people infected by SARS-CoV-2 at work, 93 (21.8%) presented clinical manifestations consistent with COVID-19, and 83 (19.4%) underwent routine workplace screening. The majority of those interviewed, 361 (84.5%), reported symptoms at the time of the positive test; the most frequent symptom was fever (n.207; 57.3%), followed by alteration/loss of smell and/or taste (n.173; 47.9%), asthenia (n.154; 42.6%), cough (n.141; 39.1%), and widespread pain (n.135; 37.4%). In most cases, multiple symptoms were present. There was no statistical difference regarding age or gender in the group reporting symptoms and those without clinical manifestations. Of note, 20 (4.7%) workers, 11 females and 9 males, were admitted to the hospital during the acute illness, with a significant difference in the mean age between the groups hospitalized and not hospitalized (U = 1973.50, *p* < 0.001). Table 2 shows the main variables which were investigated as linked to the acute condition of the infection.

### 3.3. Health Status at 3Months

#### 3.3.1. Residual Symptoms and Sleep Quality

Of the participants, 145 (33.8%) reported multiple residual symptoms at the time of the interview. The most frequent symptom was widespread pain (21%) and asthenia, present in 14% of cases. The other symptoms were impairment of smell and/or taste (20%), difficulty breathing (19%), brain fog (8%), gastrointestinal disorders (7%), cough (5%), headache (5%), ocular disorders (5%), other clinical manifestations, such as paresthesias, skin disorders, anxiety/insecurity, loss of appetite, melancholy, extrasystoles or tachycardia, dizziness, hair loss, or irritation of the upper airways, (12%). Significant differences were found between male and female workers, with females more likely to report symptoms at the time of interview (χ^2^ (1, 422) = 10.296, *p* = 0.001). A significant difference was also found between the age distribution of the groups with and without persistent symptoms, with older workers reporting symptoms more frequently (U = 12,985.500, *p* < 0.001). When performing the analysis by splitting the sample into the professional sub-groups, a significant difference was found regarding reported persistent symptoms (χ^2^ (4, 422) = 30.836, *p* < 0.001). The Healtcare Assistant group reported more symptoms than the other groups.

Moreover, 157 (37.2%) HCWs reported a change in sleep pattern after SARS-CoV-2 infection: n.131 (83.4%) declared insomnia, n.18 (11.5%) excessive daytime sleepiness, and n.5 (3.2%) both. For n. 5 respondents, the data on the type of sleep disorders is missing. A statistically significant difference was found between males and females (χ^2^(1, 422) = 4.68, *p* = 0.02), with females reporting more sleep disturbances than males. A significant difference between the mean age according to the reported sleep quality was also found (U = 17,454.00, *p* = 0.006)

Data regarding symptoms 3 months after infection and sleep quality are reported in Table 3.

#### 3.3.2. At-Work Relationships and the Organization

After returning to work, most workers (n.341; 79.5%) did not report any changes in their relationships with colleagues. For 26 (6.1%), their relationships improved, and for 20 (4.7%), their relationships became worse. No statistically significant differences were found by gender or age. Regarding workload, 68.3% of workers (n.293) reported no changes in workload, with 15% reporting an increase (n.64) and 7.7% a decrease (n.33). Nurses were more likely to report an increase in workload (χ^2^ (8, 390) = 16.21, *p* = 0.03) and worse relationships (χ^2^ (8, 3879) = 17.21, *p* = 0.028). Table 4 shows the variables analyzed according to sex, professional sub-groups, and age.

#### 3.3.3. SF-Score PCS and MCS

The SF-12 questionnaire was administered to participants at the end of the interview to determine the perception of both the physical (PCS score) and the mental (MCS score) health of the workers at the time of the interview. The values of the two scores in the sample range from 20.2 to 64.1 for the PCS and from 13.4 to 67.1 for the MCS. The mean SF-12 score for the total sample was 50.5 for the PCS (SD ± 8.8) and 46.9 for the MCS (SD ± 10.5).

We first determined whether the two scores showed differences according to sex and age. Differences in the distribution of the two scores were found by gender, with female workers perceiving worse physical and mental health status than their male counterparts (U = 23,891.5, *p* = 0.002 and U = 24,357, *p* < 0.001, respectively). We also analyzed the gender difference for each of the professional groups. For the professional group Nurse, the PCS and MCS scores differed significantly between males and females (U = 3979, *p* = 0.04 and U = 4331, *p* = 0.02, respectively). For Healthcare Assistants group, a statistically significant difference was found between males and females only for the PCS score (U = 1341.5, *p* = 0.01) but not for the MCS score (U = 1031, *p* = 0.82). For the groups Physicians, Resident Physicians, and Others, no statistically significant differences were found between males and females regarding both MCS and PCS scores.

Considering the overall sample, there was a significant negative correlation between age and the PCS score (r =−0.210, *p* < 0.001) but not between age and the MCS score (r = −0.015, *p* = 0.86). We performed the test for each of the five professional sub-groups and a significant negative correlation was confirmed for the PCS score for the Nurse and Physician groups (r = −0.124, *p* = 0.012 and r = −0.296, *p* = 0.02, respectively).

**Figure 1:**
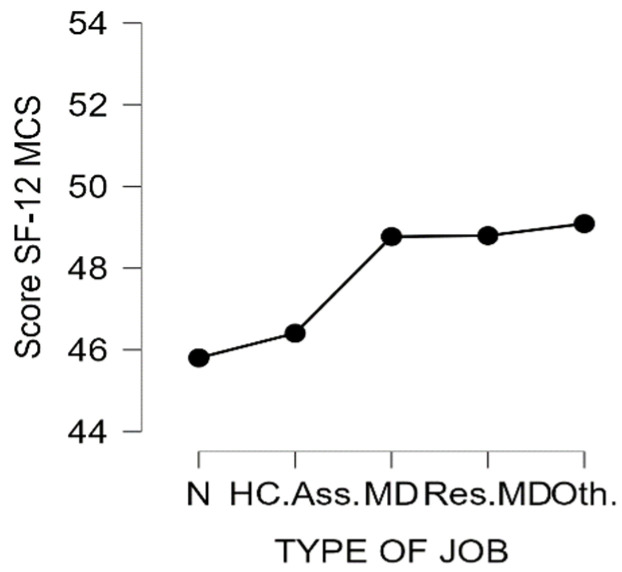
MCS score according to the type of job. N = Nurse; HC.Ass. = Healthcare Assistants; MD = Physicians; Res.MD = Resident Physicians; Oth = Others.

**Figure 2:**
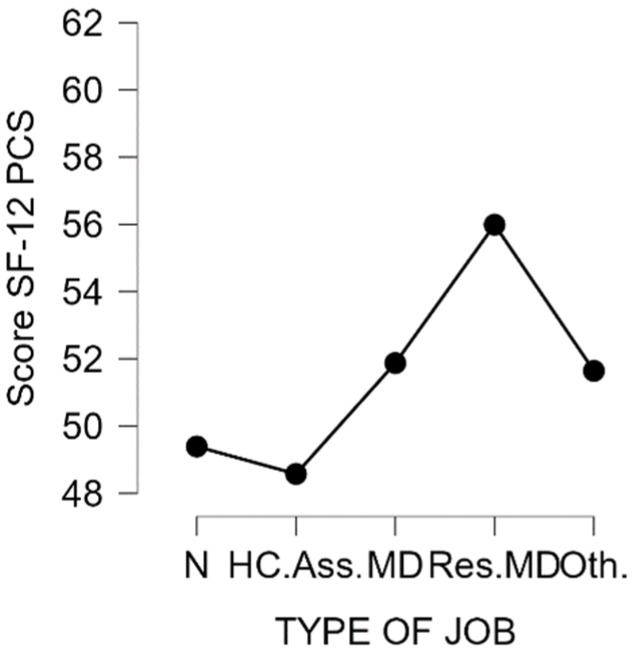
PCS score according to the type of job. N = Nurse; HC.Ass. = Healthcare Assistants; MD = Physicians; Res.MD = Resident Physicians; Oth = Others.

Then, we determined whether the professional sub-groups showed differences in the two scores. Overall, there was a significant difference in the score PCS in the five professional sub-groups (H (4) = 39.1, *p* < 0.001) but not for the MCS score (H (4) = 6.66, *p* = 0.16). For the PCS score, the difference was significant between the Nurse and Physician groups (*p* = 0.04) and for the Residents group and all other groups (*p* < 0.001). Figure 1 and Figure 2 show the distribution of the scores according to professional sub-groups.

Table 5 summarizes the mean SF-12 PCS and MCS scores according to sex and professional sub-groups.

Finally, the PCS and MCS scores were analyzed by grouping the samples according to the other variables examined in the sample. Significant differences in the means of the two scores were found in relation to sleep quality, with the group of workers reporting sleep alterations showing lower PCS (U = 25,764, *p* < 0.001) and MCS scores (U = 27,339, *p* < 0.001). Additionally, a significant difference was found between the MCS scores among workers according to their reported work relationships (H (2) = 13.24, *p* = 0.001).

## 4. Discussion

Since the very beginning of the pandemic, the health emergency has tested health-care systems worldwide, exposing and exacerbating their intrinsic weaknesses. Health-care workers have been recognized as the pivots of the system and the need to develop interventions to preserve their health at work has definitely emerged. Identifying vulnerable workers and specific workplace issues are key factors in planning interventions in the occupational health surveillance system. The findings of this study, in terms of identifying at-risk groups and the possible determinants of HCW health after COVID-19 infection, aims at broadening knowledge about the workplace wellbeing of healthcare workers as well as setting the basis for future targeted interventions led by hospital management. Contributing to HCWs’ professional wellbeing includes preserving not only workers’ health but also providing high-quality care to patients. In a post-pandemic era, in which SARS-CoV-2 is expected to spread endemically [27], coexistence with the disease passes on deep knowledge of clinical outcomes. From an occupational medicine perspective, this includes the knowledge of the relationships between clinical outcomes and returning to work in the light of the COVID-19 infection’s long-term effects. One puzzling feature of long COVID is that it affects survivors of COVID-19 from all levels of disease severity, which is consistent with our data. As shown by a previous study [28], the proportion of workers who come back to work after SARS-CoV-2 infection and still report several residual symptoms is not negligible, accounting for a third of the workers in our study. The most frequent symptom is persistent fatigue, which, according to the literature, is reported by a significant minority of patients, ranging from 13% to 33% at 16–20 weeks after symptom onset [29]. Worsening of sleep patterns was also a common reported condition in our sample, accounting for over a third of the workers interviewed. These data are in line with the literature: in a meta-analysis of over 11,000 patients, neuropsychiatric symptoms such as fatigue and sleep disorders were common 3 months after an acute SARS-CoV-2 infection [30]. Social confinement, trauma during the acute-infection, and persistent fatigue are heavily implicated in the development of sleep disorders [31]. The hypothesis regarding the influence played by age, sex, and job category on health outcomes after the SARS-CoV-2 in HCWs was confirmed from several points of view by the results of this research. Our study highlighted some differences between male and female workers, especially at 3 months after the infection. When analyzing the features of the acute illness, there were no differences between male and female workers regarding their reporting of symptoms or their hospitalization rates. Nearly two years after the pandemic onset, clinical outcomes have shown that males experience both higher severity of and fatality from COVID-19 than females [32]. In our population, probably due to the dimension of the sample and to the relatively low mean age, we did not find these differences. However, at 3 months after infection, women reported more symptoms, perceived their physical and mental health to be worse, and complained more of sleep changes than male healthcare workers. Women at returning to work seem to be more affected by the residual disturbances of COVID-19, from both mental and physical health perspectives, as shown by the PCS and MCS scores. Some studies highlighted that the long COVID syndrome could be associated with risk factors such female sex, more than five early symptoms, early dyspnea, prior psychiatric disorders, and specific biomarkers (e.g., D-dimer, CRP, and lymphocyte count), although more research is required [33,34,35]. The finding regarding worse women’s health can be also contextualized in the light of the challenging balance between work and health, with female workers generally exposed to greater work-related stress than males, which was exacerbated during the ongoing pandemic [24]. Female workers are also more vulnerable to psychosocial risks [36] and usually report a lower quality of life [37]. They usually struggle more than their male counterparts in balancing their private lives and work, especially in the healthcare setting [38]. Nevertheless, women represent the majority of workers in the healthcare setting, which is also testified to by the gender distribution of our sample. A physically and mentally healthy workforce is required to guarantee the best healthcare quality and manage clinical risk. [39]. From this point of view, work organizations should attempt to improve the aspects that may impact work–life balance in the limits of resources and healthcare needs. Interestingly, the gender difference in PCS and MCS scores is maintained when performing the analysis for the Nurse group, but not for the other professional sub-groups. This can be explained by the heterogeneity and size of the Nurse group, while the other sub-groups are more similar in their components. The professional sub-groups of our sample—namely Nurses, Healthcare Assistants, Physicians, Resident Physicians, and Other—indeed showed significant differences in their composition by age and gender distribution, and this aspect should be considered when planning interventions to boost worker wellbeing. Overall, the professional sub-groups are difficult to compare directly due to the heterogeneity of their composition. When analyzing the PCS and MCS scores between the different professions, a difference in the PCS score was found between the Nurse and Physician groups and between the Resident Physicians group and all the other groups. Regarding the latter, it should be noted that residents are young doctors in their late 20s, at the very beginning of their careers for the most part, identifying them as a specific group in which age, and probably work enthusiasm, play a relevant role. For the other difference, although the physicians are on average older than the nurses in our sample, nurses have lower PCS scores. This could be partially explained by the prevalence of females in the Nurse group, but it could also be hypothesized that nurses face a harder return to work, which affects their health perception. Nurses are in fact reporting an increase in workload, in line with previous findings reporting significantly higher workloads for nurses compared with physicians and other professional groups [40,41]. Overall, the findings from this study identify age as a major factor which impacts worker health in dealing with the acute illness. It is well known how age can impact the gravity of COVID-19 manifestations [42]. In our sample, as expected, there was a difference in the mean age between hospitalized and non-hospitalized patients. Age was also a determinant in the perception of physical status at 3 months after the infection; age may be considered not only a biological parameter but also an indicator of career length, especially for some professionals such as nurses and physicians. This finding is relevant in light of the aging of the working population [43]. The physical and mental health of aging healthcare workforces was already an overlooked public health issue before the advent of the new public health crisis due to the SARS-CoV-2 outbreak [44]. The presence of a large part of the workforce aged over 55 years exasperates the need for effective interventions to adapt the workplace to the aging working population, which is more likely to be affected by higher burdens of diseases. In Italy, a survey by the National Institute for Insurance Against Workplace Injuries (INAIL), as early as 2014, [45] revealed a deterioration in the perception of individual health status directly related to age, with the share of workers declaring very good health status varying from 49% among the workers between 16 and 24 years of age to 14% among those aged between 55 and 64, with a close correspondence between age and the trend of accidents at work. An appropriate active-aging program could provide a motivational stimulation among older workers, resulting in an improvement in workers’ psychophysical wellbeing and consequent reductions in absenteeism, accidents, and limitations at work [46,47]. The offering of health screenings to monitor the health of workers and encourage healthy workplace habits and good practices and promote active living, healthy eating, stress management, and work-life balance initiatives is expected to play a role in maintaining the health and safety of aging workers [48]. Planning such interventions may be difficult, given the heavy workloads of healthcare workers. Effective interventions should also address work organization (e.g., the distribution of night shifts and the rotation in multiple settings with different workloads) and include occupational physicians’ role in promoting healthy habits, which are effective in preserving good health status and work ability [49]. Furthermore, other needed interventions are those which can improve relationships between colleagues. Worse relationships at work, in fact, are linked to lower mental health perception scores. Social support was found as the main factor which protected the mental wellbeing of workers during the pandemic [6,36,50], and our findings support this aspect. Intervention to boost peer collaboration should be the main target for stakeholders in order to protect the mental health of workers [51,52].

This study has several limitations. Firstly, it is a cross-sectional study, thus the strength of the possible causal inferences on the relationships between the variables is limited. Secondly, some variables, such as the relationship with colleagues, were investigated through non validated tools. Thirdly, the study analyzed the outcomes in a nonvaccinated sample and did not consider data regarding the previous medical history of the participants. A further analysis of the data will address the relationships between previous medical conditions and post-infection outcomes. However, some points of strength deserve to be highlighted: data regarding the return to work of healthcare workers after the infection are missing, and to our knowledge, it is the first study investigating the perception of physical and mental health in this population 3 months after SARS-CoV-2 infection. It also identified target groups (females, older workers, nurses) who can be more vulnerable to the physical and mental burden of an acute illness such as COVID-19. In addition, our study provides some interesting insights into the general health status after COVID-19 of a relatively young and working population, showing the relevant residual health conditions (fatigue, sleep disorders). 

## 5. Conclusions

Over a third of healthcare workers at three months after SARS-CoV-2 infection reported residual symptoms, especially fatigue and sleep disorders. Females and older workers are vulnerable categories, complaining of poorer physical and mental health after the infection. The heterogeneity of age and sex of professional groups among healthcare workers is a challenge when planning interventions to improve their occupational health, in both the physical and mental spheres, where nurses seem to be a particularly complex and possibly vulnerable group. Tailoring specific programs to professional sub-groups, such as health promotion and healthy aging plans or initiatives which could improve professional relationships, might be valuable to buffer and mitigate the physical and mental outcomes of the healthcare worker population in the post-pandemic era.

## Figures and Tables

**Table 1:** Main variables analyzed in the study, for the general sample and for professional sub-groups.

	General	Males	Female	Nurse (186, 43.6%)	Healthcare Assistants (104, 24.4%)	Physicians (51, 11.9%)	Resident Physicians (54, 12.6%)	Other (32, 7.5%)
**Age (n** **±** **sd)**	45.3 ± 11.9 (range 23–67)	45.6 ± 13.1 (range 24–67)	45.2 ±11.3 (range 23–66)	45.2 ± 11.1 (range 23–66)	49.9 ± 9 (range 29–66)	52.8 ± 11.4 (range 33–67)	30 ± 1.8 (range 27–36)	45.6 ±12.6 (range 24–67)
**Males (n, %)**	143 (33.3)	--	--	48 (25.8)	26 (25)	37 (72.5)	23 (42.6)	9 (28.1)
**Females (n, %)**	284 (66.7)	--	--	138 (74.2)	78 (75)	14 (27.5)	31 (57.4)	23 (71.9)

**Table 2:** Acute symptoms and hospitalization according to sex, professional sub-groups and age.

	Acute Symptoms	*p*-Value	Hospitalization	*p*-Value
**General**	361 (84.5)		20 (4.7)	
**Sex**		0.094		0.26
Male	115 (80.4)		9 (6.3)	
Female	246 (86.6)		11 (3.9)	
**Professional sub-groups**		0.748		0.106
Nurse	158 (84.9)		8 (4.3)	
Healthcare assistants	86 (82.7)		4 (3.8)	
Physicians	46 (90.2)		6 (11.8)	
Resident physicians	44 (81.5)		2 (3.7)	
Other	27 (84.4)		0	
Mean age	45.5 ± 11.9	0.58	55.2 ± 10.5	<0.001 ***

Significant results are indicated with *. *** *p* < 0.001.

**Table 3:** Residual symptoms and changes in sleep quality 3 months after SARS-CoV-2 infection.

	Residual Symptoms	*p*-Value	Sleep Quality	*p*-Value
**General**	145 (34.4)		157 (37.2)	
**Sex**		0.001 **		0.002 **
Male	34 (23.9)		42 (29.8)	
Female	111 (39.6)		115 (40.9)	
**Professional sub-groups**		<0.001 ***		0.21
Nurse	74 (40.2)		77 (41.4)	
Healthcare assistants	48 (46.6) ^†^		40 (38.8)	
Physicians	9 (18.4)		14 (28.5)	
Resident physicians	5 (9.3)		14 (26.4.)	
Other	9 (28.1)		12 (38.7)	
Mean age	50.1 ± 10.4	<0.001 ***	47.6 ±11.2	0.006 **

Significant results are indicated with *. ** *p* < 0.01, *** *p* < 0.001; ^†^ Significant difference between observed and expected frequency at post hoc analysis.

**Table 4:** At-work relationships and workload 3 months after the infection according to sex, professional sub-groups, and age.

	Improved Relationships	Worse Relationships	No Change	*p*-Value	Increased Workload	Decreased Workload	No Change	*p*-Value
**General**	26 (6.7)	20 (5.2)	341 (88.1)		64 (16.4)	33 (8.5)		
**Sex**				0.07				0.25
Male	6 (4.6)	3 (2.3)	122 (93.1)		16 (12.2)	11 (8.3)	105 (79.5)	
Female	20 (7)	17 (6.6)	219 (85.4)		48 (18.6)	22 (8.5)	188 (72.9)	
**Professional sub-groups**				0.028 *				0.03 *
Nurse	14 (8.2)	16 (9.4) ^†^	140 (82.4)		40 (23.3) ^†^	16 (9.39)	116 (67.4)	
Healthcare assistants	8 (8.5)	3 (3.2)	83 (88.3)		13 (13.7)	6 (6.3)	76 (80)	
Physicians	1 (2.2)	0	44 (97.8)		4 (8.9)	3 (6.7)	38 (84.4)	
Resident physicians	1 (2.1)	0	47 (97.9)		2 (4.2)	4 (8.3)	42 (87.5)	
Other	2 (6.7)	1 (3.3)	27 (90.0)		5 (16.7)	4 (13.3)	21 (70.0)	
**Mean age**	45.0 ± 12.5	45.0 ± 12.0	45.0 ± 12	0.19	45.1 ±11.9	43.6 ± 12.6	45.1 ± 11.9	0.45

Significant results are indicated with *. * *p* < 0.05; ^†^ Significant difference between observed and expected frequency at post hoc analysis.

**Table 5:** Distribution of SF-12 PCS and MCS according to sex and professional sub-groups.

	Score SF-12 PCS	*p*-Value	Score SF-12 MCS	*p*-Value
**General**	50.5 ± 8.8 (range 20.2–64.1)		46.9 ± 10.5 (range 13.4–67.1)	
**Sex**		0.02 *		<0.001 ***
Male	52.5 ± 7 (range 20.2–62.6)		49.4 ±9.5 (range 19.8–66.5)	
Female	49.5 ± 9.4 (range 21–64.1)		45.7 ± 10.8 (range 13.4–67.1)	
**Professional sub-groups**		<0.001 ***		0.155
Nurse	49.4 ± 9 (range 20.2–64.1)	N-MD 0.041 *	45.8 ± 10.7 (range 13.4–67.1)	
		N-Res.MD < 0.001 ***		
Healthcare assistants	48.6 ± 10.1 (range 21.6–63.4)	HC.Ass.-Res.MD < 0.001 ***	46.4 ±11.2 (range 20.6–62.9)	
Physicians	51.9 ± 7.1. (range 27.7–61.7)	MD-Res.MD < 0.001 ***	48.8 ± 10.4 (range 19.8–66.2)	
Resident physicians	56 ± 4.4 (range 30–62.5)		48.8 ± 9 (range 25.7–60.8)	
Other	51.6 ± 6.6 (range 30–60.8)	Oth.-Res.MD < 0.001 ***	49.1 ± 8.7 (range 28.4–66.5)	

Significant results are indicated with *. * *p* < 0.05, *** *p* < 0.001.

## Data Availability

Data supporting the reported results are available on request.

## Supplementary Materials

The following supporting information can be downloaded at: https://www.mdpi.com/article/10.3390/ijerph19116811/s1, Supplementary Materials: Questionnaire administered to HCWs.
