# Supplementary material for: Return to Work of Healthcare Workers after SARS-CoV-2 Infection: Determinants of Physical and Mental Health
Source: Int J Environ Res Public Health. 2022 Jun 2;19(11):6811. doi: 10.3390/ijerph19116811 (PMC9180816; doi:10.3390/ijerph19116811)
Supplement: Supplementary file 1 [file ijerph-19-06811-s001.zip › ijerph-1720116-supplementary.pdf]

S1-**Supplementary Material**

Survey on health care workers (HCWs) with positive nasopharyngeal swab for SARS-CoV-2

**Personal data**	
Date of the interview	I__I__I / I__I__I/ I__I__I
Date of birth	I__I__I / I__I__I/ I__I__I
Sex	M I__I F I__I
Place of residency (PR)	
Place of current domicile (if different from residency) (PR)	
Type of HCW	◎ Physician<br>◎ Resident physician<br>◎ Nurse<br>◎ Nurse student<br>◎ Healthcare Assistant<br>◎ Midwife<br>◎ Rehabilitation professional<br>◎ Technician (specify)…….<br>◎ Clerk<br>◎ Other………
Operative Unit	____________________

**Follow-up on HCWs health status**		
1	Date of the first positive swab	Date …….
2	Why did you perform the swab? (multiple answers are possible)	◎ Contact with COVID-19 infected patient<br>◎ Contact with COVID-19 infected colleague<br>◎ Contact with a COVID-19 case outside the working setting (e.g. family members<br>◎ COVID-like<br>◎ Screening sierology for COVID antibodies was positive<br>◎ Return to work after influenza like symptoms.<br>◎ Changing ward from COVID area to non COVID area.
3	Where did you perform the swab?	◎ Biological Risk ambulatory (Infective Diseases Unit)

		◎ Occupational Medicine Service<br>◎ Emrgency Department (of which hospital?)<br>◎ At home or other facilities (Public Health, General Medicine services)
4	In the week before you tested positive, where did you work?	◎ COVID Area<br>◎ Non-COVID Area<br>◎ Other…………
5	Did you present any COVID symptoms?	◎ NO<br>◎ YES (starting from date /…../….)
6	Only if you answered yes to question 5, which symptoms did you have?	◎ Fever>37.5°<br>◎ Cough<br>◎ Shortness of breathing<br>◎ Diarrhea<br>◎ Asthenai<br>◎ Taste alterations or loss<br>◎ Smell alterations or loss<br>◎ Eyes disturbs (e.g. conjunctivitis)<br>◎ Other________
7	Have you been hospitalized due to infection?	◎ NO<br>◎ YES, specify date of admission, length of stay, Hospital and ward.
8	Did take any medication at home for the infection?	◎ NO<br>◎ YES, which and for how long?
9	Do you live with others?	◎ NO<br>◎ YES, how many?
10	Only if the answer to question 9 was YES, did any of your cohabitants develop any COVID-like symptoms since you knew you were positive?	◎ NO<br>◎ YES. For each cohabitant please specify if he/she was tested positive.
11	Did you perform the swab to certify the end of infection?	◎ NO

		◎ YES. If yes, please specify how many and for each one date and setting.
12	Did you perform any control visit or blood/instrumental tests after the disease?	◎ NO<br>◎ YES. If yes, please specify what, date of execution and results.
13	Only if you answered YES to question 5. Did you present any symptom right now?	◎ NO<br>◎ YES. If yes, please specify.
14	Did you return to work?	◎ NO<br>◎ YES. If yes, please specify the date you came back to work.
15	If the answer to question 14 is YES, since you returned to work…	a. To what service was you assigned? _______<br>b. Were your tasks modified?<br>c. The relationships with your colleagues are:<br>- improved<br>-worse<br>-not modified<br>d. Workload is<br>-not modified<br>-augmented<br>-reduced<br>e. Do you want to point out any other changes happened?
16	After the infection, is the quality of your sleep modified?	◎ NO<br>◎ YES. If yes, please specify in what way.

SF-12 Short Form Health Survey

The SF-12 questionnaire administered to the sample is the one developed by Jenkinson et al. (Jenkinson, C.; Layte, R. Development and Testing of the UK SF-12. *J. Health Serv. Res. Policy* **1997**, *2*, 14–18, doi:10.1177/135581969700200105.) and can be found online in the original English version.

The synthesis of the scores allows to construct two indices of the state of health: one concerning the physical state (Physical Component Summary - PCS), the other the psychological state (Mental Component Summary - MCS). Very low levels of PCS (under 20 points) correspond to substantial limitations in self-care and physical, social and personal activity, significant physical pain, frequent fatigue, health status perceived as poor. Very low levels of MCS mean frequent psychological distress and significant social and personal disability due to emotional problems. Very high levels of PCS correspond to a condition of no physical limitation, disability or decrease in general well-being, high vitality, health perceived as excellent. Very high levels of MCS show positive psychological attitudes, absence of psychological distress and limitations in social and personal activities due to emotional problems.
